# Psychological Characteristics of Students with Passion for Studying

**DOI:** 10.3390/bs14060453

**Published:** 2024-05-28

**Authors:** Paweł Larionow, Agnieszka Gabryś

**Affiliations:** 1 Faculty of Psychology, Kazimierz Wielki University, 85-064 Bydgoszcz, Poland; 2 Faculty of Education and Psychology, Institute of Pedagogy, Maria Curie-Skłodowska University, 20-612 Lublin, Poland; agnieszka.gabrys@mail.umcs.pl

**Keywords:** academic burnout, harmonious passion, mental health, obsessive passion, person-centered approach, psychological characteristics, students, studying, variable-centered approach, well-being

## Abstract

Passion for studying can be considered a significant factor that promotes well-being and mental health in students. This study aimed to examine whether the psychological characteristics of students with a passion for studying differed from those of students without one. To compare these two groups, we used a set of different psychological variables (e.g., academic burnout and vitality), as well as integrated both person-centered (i.e., group comparison research) and variable-centered (i.e., correlational analysis) approaches. During classes, one hundred and fifty-four students from a Polish university completed a comprehensive set of short self-report questionnaires online on different psychological characteristics, including variables related to studying (i.e., passion for studying, academic burnout, and general academic self-efficacy), psychopathology symptoms, perceived stress and somatic complaints, as well as personal resources (vitality, resilience, self-esteem, and optimism). We noted multiple statistically significant differences in psychological characteristics between the two studied groups of students. Thus, harmonious passionate students tended to have more favorable psychological characteristics within variables related to studying, mental or somatic health symptoms, and personal resources compared to the non-passionate students. A harmonious passion for studying seems to have potential health-promoting and health-protecting effects, whereas a lack of passion for studying may lead to less favorable outcomes.

## 1. Introduction

Academic education is a demanding process filled not only with achievements but also challenges and difficulties which may lead to students’ academic burnout [1]. Academic burnout (also known as learning burnout [2]) is a state of long-lasting negative emotions related to the process of studying [3]. Although more than half of university students tend to have high levels of academic burnout (see [4] for review), studying is considered a passion for many students.

### 1.1. The Dualistic Model of Passion

Passion is a person’s strong inclination toward a particular activity, object, or a person that one loves (or at least likes very much), highly values, and invests energy and time in, as it is postulated in the Dualistic Model of Passion [5]. A student has a passion for studying when they enjoy (or love) their studies, treat them as part of their identity, devote time to learning, and define it as their passion. The Dualistic Model of Passion [5] describes two types of passion: harmonious passion and obsessive passion.

Harmonious passion is related to the autonomous internalization of a given activity in a person’s identity [6]. Within harmonious passion, a person remains in control of the activity and decides when to engage in this activity and when not to [6]. Harmonious passion is in harmony with other aspects of a person’s life, producing favorable outcomes (e.g., positive emotions, flexible engagement, and high performance) [5]. Obsessive passion, in contrast, results from the controlled internalization of activity in a person’s identity [6]. A person with obsessive passion may experience an uncontrollable urge to participate in an activity [6,7]. Consequently, the passion for the activity begins to control the person with obsessive passion [7]. Having obsessive passion complicates daily functioning and therefore often provides fewer desirable outcomes, including low well-being and high ill-being levels [5,6,8]. A meta-analytic review by Curran et al. [9] indicated that obsessive passion was positively correlated with both positive and negative intrapersonal outcomes, suggesting that the effects of obsessive passion can be equivocal. In contrast, harmonious passion was positively linked to mainly positive intrapersonal outcomes [9].

In order to assess passion, Vallerand et al. [7,10] developed the Passion Scale (PS). This scale consists of 17 items, with six items measuring harmonious passion (e.g., “*This activity is in harmony with the other activities in my life*”) and the other six items assessing obsessive passion (e.g., “*This activity is so exciting that I sometimes lose control over it*”) [7], p. 760. Five more items measure the passion criteria (e.g., “*I spend a lot of time doing this activity*”) [7], p. 760, which are used for determining the presence of a passion [7,10].

### 1.2. Literature Review on Passion for Studying

In samples of students, previous studies indicated that harmonious passion was positively related to well-being, good self-regulated learning [11] as well as to positive intrapersonal characteristics in general (e.g., more adaptive emotion regulation strategies [12]). It was also related to lower levels of academic burnout [13], less academic procrastination [14], and fewer other negative psychological outcomes (e.g., psychological distress and negative affect) [14,15]. As for obsessive passion, it was positively related to academic burnout [13] and negative affect while engaging in an activity [15] as well as with other negative interpersonal characteristics (e.g., less adaptive emotion regulation strategies [12]). Obsessive passion was negatively related to well-being and self-regulated learning among students [11].

Controlling for autonomous and controlled motivation, both harmonious passion and obsessive passion predicted higher vigor and efficacy in university students [16]. Overall, both dimensions of passion were associated with higher academic engagement and lower academic burnout; however, the effects of harmonious passion were stronger than the effects of obsessive passion [16]. In the education domain, Paquette et al. [17] showed that harmonious passion was related to higher satisfaction with studies as well as subjective and objective performance in studies. In the health domain, harmonious passion was associated with higher levels of subjective vitality and lower levels of common somatic (or physical) symptoms (e.g., headache) [17]. In contrast, obsessive passion was mainly related to less favorable outcomes [17]. Overall, Paquette et al. [17] indicated that harmonious passion acted as a contributing factor to high levels of global resilience in the face of a stressful event, which was also empirically supported in the experimental study by Vallerand et al. [18] devoted to examining the role of passion for studying in psychosomatic responses to a stressful situation. Considering the above, harmonious passion seemed to play a more influential role in various aspects of students’ life than obsessive passion. Overall, these findings highlight the importance of passion for studying in students’ academic life, especially among students with harmonious passion.

### 1.3. Examination of Passion for Studying: The Variable-Centered and Person-Centered Approaches

In most previous studies on passion, a variable-centered approach (based mainly on correlational analysis) was used. However, this type of analysis does not allow taking into account the coexistence of harmonious passion and obsessive passion in a person’s identity [19]. A person-centered approach [20] can be implemented to address the indicated limitations. In past work on passion, this approach was used for a relatively small number of studies, mainly devoted to passion for work (e.g., Li et al. [21], Mudło-Głagolska [22]). There exist only a few studies on passion for studying based on a person-centered approach (e.g., Bélanger and Ratelle [23] and Mudło-Głagolska and Larionow [24]). For example, Bélanger and Ratelle [23] examined passion profiles among students using latent profile analysis, with four profiles identified: (1) High (high harmonious passion and obsessive passion), (2) Moderate (moderate harmonious passion and obsessive passion), (3) Low (low harmonious passion and obsessive passion), and (4) Optimal (high harmonious passion and low obsessive passion). Among these profiles, students with high passion scores (High and Optimal profiles) showed the best academic functioning, whereas students with low passion scores (Low profile) reported the worst academic functioning [23]. A similar latent profile analysis was applied in a Polish study by Mudło-Głagolska and Larionow [24], who came to the same conclusions as Bélanger and Ratelle [23] regarding the worst academic functioning in students with low passion scores. Overall, the above-mentioned studies showed that a passion for studying was associated with better academic functioning.

We consider this comprehensive statistical approach using latent profile analysis highly beneficial, with its use being especially relevant for research. However, this approach may be less attractive in practice (e.g., in evaluating and monitoring students’ functioning or in implementing effective psychological support for students in the academic context); therefore, we believe that simpler methodological approaches could be beneficial. To exemplify, passion for studying can be revealed based on the passion criteria scores of equal and larger than 5 in the PS [7]. For instance, based on these criteria, a Polish study by Zinczuk-Zielazna [15] has shown that approximately one-half of students (56.2%) were passionate about studying. Compared to students without passion for studying, the passionate were characterized by statistically significant higher levels of positive affect, quality of interpersonal relationships, and internal motivation for studying as well as higher grade point average, along with lower levels of negative affect and external motivation [15]. Even though the predominant passion profile (harmonious passion over obsessive passion or vice versa, or mixed passion) among students passionate about studying was not examined in Zinczuk-Zielazna’s study [15], we anticipated similar results in our research, predicting more favorable psychological characteristics among students with passion for studying compared to non-passionate about studying.

### 1.4. The Current Study

This study aimed to examine whether students with a passion for studying differ from students without a passion for studying in their psychological characteristics. In order to explore this comprehensively, we addressed different psychological variables related to studying (i.e., academic burnout, general academic self-efficacy), mental (i.e., psychopathology symptoms, and perceived stress) or somatic (i.e., somatic complaints) health symptoms, and personal resources (i.e., vitality, resilience, self-esteem, and optimism).

Based on theory and the above-described studies, we were interested in examining our variables of interest chosen for this study. We feel that psychological variables related to studying (i.e., academic burnout, general academic self-efficacy) would be of high relevance in our research on passion for studying, because these variables are thematically strongly interplayed. For example, general academic self-efficacy represents the global belief in one’s ability to work well within the whole academic process [25,26], hence both harmonious passion and obsessive passion seem to be positively associated with academic self-efficacy. Assessing general health variables, including mental (i.e., anxiety and depression symptoms as well as perceived stress) and common somatic/physical symptoms (e.g., backache, stomachache), is valuable to understand whether passion for studying plays a health-promoting role in students. For example, as perceived stress represents the extent to which situations in one’s life are subjectively appraised as stressful [27], we strived to reveal whether there were differences between students with a passion for studying and students without one. Also, evaluating personal resources is helpful to understand whether passion for studying is interrelated with personal resources, enhancing their synergetic effects, or acts as an isolated phenomenon, which is not related to personal resources. With this in mind, we were interested in assessing vitality as a trait (refers to a positive sense of aliveness and energy in the organism [28], resilience (refers to the ability to bounce back or recover from stressful situations [29]), self-esteem (refers to the general attitude toward oneself [30]) and optimism (refers to a sense of positive attitude to life with expectations that good things will happen [31]).

In the current research, we integrated both the person-centered approach (i.e., identifying and examining students with a passion for studying and non-passionate students) and the variable-centered approach (i.e., correlational analysis), focusing on the person-centered approach. We predicted that (1) approximately one-half of students would have a passion for studying, and (2) students with a passion for studying would tend to report lower levels of academic burnout, mental or somatic health symptoms as well as higher levels of general academic self-efficacy, and personal resources, compared to students without a passion for studying.

## 2. Materials and Methods

### 2.1. Procedure

This study was conducted according to the Declaration of Helsinki Ethical Principles. The institutional review board of the Faculty of Psychology of Kazimierz Wielki University (No. 2/14 March 2023) approved the current study.

Our students were invited to complete an online anonymous survey via Google Forms during classes at the Maria Curie-Skłodowska University (Lublin, Poland). Participants agreed to take part in the survey without remuneration and provided their written informed consent. In this cross-sectional study, our participants filled out a demographic form and answered a set of short self-report questionnaires (refer to Section 2.3 for details).

### 2.2. Participants

The participants were 154 (143 females and 11 males) social science students (predominantly pedagogy students), attending basically in the first and second years at the Maria Curie-Skłodowska University. The mean age of students was 21.82 (*SD* = 2.45) years, with ages ranging from 18 to 40 years. Among the participants, 92 students (59.74%) had a secondary education degree, whereas 62 students (40.26%) had a higher university degree.

### 2.3. Measures

Our participants filled out digitally written informed consent and a demographic form, with questions about their sex/gender, age, education level as well as field of this study and a year of study. After completing this information, our students filled out a series of the below-described self-report questionnaires.

#### 2.3.1. The Passion Scale (PS)

The PS [7,10], in its Polish version [32] adapted for measuring passion for studying in our previous study [12,24], was used. The scale has 17 items and three subscales: a six-item harmonious passion subscale (e.g., “*My studies are in harmony with the other activities in my life*”), a six-item obsessive passion subscale (e.g., “*My studies are so exciting that I sometimes lose control over them*”), and a five-item passion criteria subscale (e.g., “*I spend a lot of time doing my studies*”). Responses are given on a seven-point response scale from 1 (“strongly disagree”) to 7 (“strongly agree”), with higher scores indicating higher levels of these subscale scores. Based on past work [7], we used an average passion criteria score of 5 as a cut-off score, indicating a presence of passion for studying.

#### 2.3.2. The Oldenburg Burnout Inventory (OLBI)

The OLBI [33], in its Polish version [34] adapted for measuring academic burnout in our previous study [24], was used. The OLBI has 16 items, with two eight-item subscales for measuring the two academic burnout dimensions: disengagement (e.g., “*It happens more and more often that I talk about my studies in a negative way*”) and exhaustion (e.g., “*During my studies, I often feel emotionally drained*”) [33]. Responses are given on a four-point response scale from 1 (“strongly agree”) to 4 (“strongly disagree”), with higher scores indicating higher levels of these two academic burnout dimensions.

#### 2.3.3. The General Academic Self-Efficacy Scale (GASE)

The GASE [25], translated into Polish, was used to assess general academic self-efficacy, which refers to the global belief in one’s ability to work well within the whole academic process [25,26]. The GASE is a four-item questionnaire (e.g., “*I will remain calm in my exam because I know I will have the knowledge to solve the problems*”), with a five-point response scale from 1 (“strongly disagree”) to 5 (“strongly agree”) [25]. Higher scores indicate higher general academic self-efficacy.

#### 2.3.4. The Patient Health Questionnaire-4 (PHQ-4)

The PHQ-4 [35], in its Polish version [36], was used to assess anxiety and depression symptoms. The PHQ-4 is a four-item questionnaire that includes two two-item subscales: anxiety (e.g., “*Feeling nervous, anxious, or on edge*”) and depression (e.g., “*Little interest or pleasure in doing things*”) [35]. The participants rate how often they experienced anxiety and depression symptoms in the past two weeks, using a four-point response scale from 0 (“not at all”) to 3 (“almost every day”), with higher scores indicating higher levels of these symptoms.

#### 2.3.5. The Giessen Subjective Complaints List (GBB-8)

The GBB-8 [37], in its Polish version [38], was used to assess somatic symptoms in four categories: exhaustion (i.e., “*Being easily exhausted*”; “*Tiredness*”), gastrointestinal (i.e., “*Feeling bloated or distended*”; “*Stomachache*”), musculoskeletal (i.e., “*Backache*”; “*Neck or shoulder pain*”), and cardiovascular complaints (i.e., “*Palpitations or heart pounding*”; “*Dizziness*”) [37]. The GBB-8 is an eight-item questionnaire, with four two-item subscales, each representing an individual category of somatic symptoms. The GBB-8 uses a five-point response scale from 0 (“not at all”) to 4 (“very much”), with higher scores indicating higher levels of these symptoms.

#### 2.3.6. The Subjective Vitality Scale (SVS)

The SVS [28], in its Polish version [39], was used for assessing vitality as a trait. The SVS is a five-item questionnaire (e.g., “*I feel alive and vital*”), with a seven-point response scale from 1 (“not true at all”) to 7 (“very true”) [28]. Higher scores indicate higher vitality.

#### 2.3.7. The Brief Resilience Scale (BRS)

The BRS [29], in its Polish version [40], was used to assess resilience as a trait. The BRS is a six-item questionnaire (e.g., “*I tend to bounce back quickly after hard times*”), with a five-point response scale from 1 (“strongly disagree”) to 5 (“strongly agree”) [29]. Higher scores indicate higher resilience.

#### 2.3.8. The Rosenberg Self-Esteem Scale (SES)

The SES [30], in its Polish version [41], was used to assess self-esteem. The SES is a ten-item questionnaire (e.g., “*On the whole, I am satisfied with myself*”), with a four-point response scale from 1 (“strongly agree”) to 4 (“strongly disagree”) [30]. Higher scores indicate higher self-esteem.

#### 2.3.9. The Optimism–Pessimism Short Scale-2 (SOP2)

The SOP2 [31], translated into Polish, was used to assess optimism. The SOP2 is a two-item questionnaire (e.g., “*Optimists are people who look to the future with confidence and who mostly expect good things to happen. How would you describe yourself? How optimistic are you in general?*”), with a seven-point response scale from 1 (“not at all optimistic” or “not at all pessimistic”) to 7 (“very optimistic” or “very pessimistic”) [31]. Higher scores indicate higher optimism.

#### 2.3.10. The Perceived Stress Scale (PSS-4)

The PSS-4 [27], in its Polish version [42], was used to assess perceived stress over the past month. The PSS-4 is a four-item questionnaire (e.g., “*In the last month, how often have you felt that you were unable to control the important things in your life?*”), with a five-point response scale from 0 (“never”) to 4 (“very often”) [27]. Higher scores indicate higher stress.

### 2.4. Statistical Analysis

*JASP* v. 0.18.3 was used for all statistical analyses. We calculated descriptive statistics, internal consistency reliability with Cronbach’s alpha coefficients, and Pearson correlations between the study variables. We compared psychological characteristics between students without a passion for studying and students with a passion for studying using the independent samples *t*-test. For this test, we reported Cohen’s *d* effect size.

## 3. Results

### 3.1. Descriptive Statistics

We presented descriptive statistics and internal consistency reliability for the study variables in Table 1. Cronbach’s alpha coefficients for all the study variables were acceptable to good (from 0.69 to 0.89), except the GBB-8 Cardiovascular complaints subscale, which showed low internal consistency reliability with Cronbach’s alpha of 0.58. This low reliability was presented in the Polish validation study of the GBB-8 [38], and this should be considered as something other than an artefact in the current study.

### 3.2. Differences in Psychological Characteristics between Students without Passion for Studying and Students with Harmonious Passion for Studying

Based on the passion criteria scores of equal and larger than 5, we allocated two groups of students. The first group involved students who reported passion criteria scores of less than 5, and these students were classified into the group of students without passion for studying (*n* = 89). The second group involved students who reported passion criteria scores of equal and larger than 5, and these students were classified into the group of students with a passion for studying (*n* = 65). In this group, 62 students had predominant harmonious passion for studying scores over obsessive passion for studying scores, 2 students had equal harmonious passion and obsessive passion scores (mixed passion for studying), and 1 student had a predominant obsessive passion score over the harmonious passion score. We decided to remove two students with mixed passion for studying, and a student with obsessive passion for studying from our comparative analysis since the majority of students with harmonious passion for studying were presented in this dataset. Then, we checked whether the psychological characteristics of students without passion for studying (*n* = 89) were different from the psychological characteristics of students with harmonious passion for studying (*n* = 62).

In Table 2, we presented the analysis of differences between the psychological characteristics of students without a passion for studying and the ones of students with harmonious passion for studying.

Our analysis indicated that, compared to students without a passion for studying, students with harmonious passion for studying had lower levels of academic burnout (the disengagement and exhaustion dimensions) and depression symptoms along with higher levels of general academic self-efficacy, vitality, resilience, and self-esteem.

Pearson correlations between the study variables are presented in Table 3.

Harmonious passion showed statistically significant positive correlations with obsessive passion, passion criteria, general academic self-efficacy, vitality, resilience, and optimism, along with statistically significant negative correlations with academic burnout (the disengagement and exhaustion dimensions), anxiety and depression symptoms, several somatic symptoms, and stress. No statistically significant correlations were noted between harmonious passion and gastrointestinal/cardiovascular complaints, or self-esteem. In contrast, obsessive passion demonstrated statistically significant positive associations with harmonious passion and passion criteria, as well as negative ones with the disengagement dimension and self-esteem. The correlational analysis revealed a lot of statistically insignificant links between obsessive passion and other variables of interest (see detailed results in Table 3).

## 4. Discussion

Our main aim in this study was to examine whether a wide range of psychological characteristics differ between the passionate and the non-passionate about studying. In this research, we included variables related to studies, mental health, and somatic symptoms, as well as personal resources. Also, we integrated both person-centered and variable-centered approaches.

### 4.1. A Person-Centered and Variable-Centered Approach Perspectives

Using a person-centered approach, we distinguished two groups of students (based on the passion criteria scores of equal and larger than 5): the first group involved the passionate about studying (*n* = 65), and the second one involved the non-passionate about studying (*n* = 89). Therefore, in our sample, approximately 42.21% of students had a passion for studying. This result was somewhat lower than the one obtained in the Polish study by Zinczuk-Zielazna [15], who showed that 56.2% of students were passionate about studying. Also, in our study, we revealed that among the passionate about studying (*n* = 65), the vast majority (*n* = 62; 95.38%) had harmonious passion for studying, whereas 2 students (3.08%) had mixed passion for studying (equal harmonious passion and obsessive passion scores), and 1 student (1.54%) had predominant obsessive passion for studying (obsessive passion scores exceeding harmonious passion scores). Overall, these results indicated that mixed passion and obsessive passion for studying were quite rare in this dataset. On the one hand, the low proportion of a mixed or obsessive passion for studying seems optimal, as such students tend to have less favorable academic functioning than harmonious passionates [23,24]. On the other hand, students without a passion for studying tend to function academically worse than students with any passion for studying (regardless of the passion type) [15,23,24]. In short, our data on the prevalence of passion for studying reflected two extremities: a positive side presented nearly an absence of mixed or obsessive passions for studying, whereas a negative one showed a relatively high proportion of non-passionates for studying.

In our study, we compared the students, non-passionate about studying, and the ones with harmonious passion for studying within a wide range of psychological variables. Consistently with the past literature [13,19,24], we revealed that students with harmonious passion for studying tended to have better academic functioning (i.e., lower academic burnout and higher general academic self-efficacy), better mental health (i.e., lower depression symptoms), as well as higher levels of personal resources (i.e., higher vitality, resilience, and self-esteem), compared to the non-passionate about studying. Across the study variables, the highest effect sizes of the differences between the two groups were noted for the two dimensions of academic burnout (i.e., disengagement and exhaustion), indicating the strong interplay between academic burnout and passion for studying. This can be served as empirical evidence for the justification of psychological support interventions based on the development of passion for studying for students with a lack of passion for studying and/or for students with high levels of burnout. We would also like to note statistically insignificant differences in anxiety symptoms, overall levels of psychopathology symptoms, all somatic complaints, optimism, and stress between these two groups (with small effect sizes).

In general, our study showed that harmonious passion for studying was something other than an isolated or specific phenomenon that only has theoretically justified relationships with studying-related variables but demonstrates no links with other variables such as mental and somatic health or personal resources. Indeed, harmonious passion for studying was related to better mental health and higher levels of various psychological resources. This suggests that harmonious passion for studying acts as a positive psychological factor, with general health-promoting (i.e., a potential increase in the levels of personal resources) and health-protecting effects (i.e., a probable decrease in negative mental health indicators). These considerations correspond with the results of previous studies where a person-centered approach was used, supporting our findings that harmonious passionates about studying had better psychological characteristics compared to non-passionates about studying [13,19,24].

Using a variable-centered approach via correlational analysis, we supported our findings derived from the person-centered approach. In our correlational analysis, harmonious passion was statistically significantly associated with most psychological characteristics examined in this study, whereas obsessive passion was associated with only a few of them (i.e., negative correlations of obsessive passion with the disengagement dimension and self-esteem). Higher levels of harmonious passion were correlated with higher levels of personal resources as well as with lower levels of outcomes for mental and somatic health, including lower levels of academic burnout. Overall, our results regarding harmonious passion and its positive associations with favorable psychological characteristics in students are aligned with a large body of past works [11,13,14,16,17], including experimental studies on physiological responses to a stressful situation [18]. In sum, the role of harmonious passion in different aspects of people’s functioning seems more comprehensive, whereas the role of obsessive passion seems limited due to generally low levels of obsessive passion and its low prevalence within passion profiles.

### 4.2. Practical Implications of This Study

The current investigation showed that research on passion for studying which involves both person-centered and variable-centered approaches is beneficial for the theory and practice of passion. From the practical point of view, we assume that a person-centered approach allows for obtaining more specific data on the passion for studying. For example, examining passion study profile prevalence among students, and comparing them across different psychological characteristics seems a simple and useful approach, with its application leading to better applicability of the obtained results in practice.

The results of this cross-sectional study indicated that among students, harmonious passion for studying was a significant factor positively associated with a wide range of favorable psychological characteristics, with its potential health-promoting and health-protecting effects. Therefore, we believe that assessment of passion for studying should be one of the important elements in several areas of educational context: (1) screening assessments of passion profiles and their prevalence, (2) predicting mental health outcomes and providing psychological interventions taking into account students’ passion for studying, (3) preventing and minimalizing negative consequences of academic burnout, especially among students without a passion for studying, and (4) implementing psychological support programs targeted at the development of harmonious passion for studying.

### 4.3. Limitations of This Study

This was a cross-sectional study, hence, longitudinal research would be beneficial in future work to determine the directionality of the study variables. In our study, the number of female participants predominated over male ones; however, this is a typical situation among the examined group (i.e., pedagogical students). Our sample size was relatively small; however, the two compared groups were similar in size, allowing reasonable comparisons. Despite these limitations, our exploratory analysis showed the benefits of passion profile assessment, based on the passion criteria scores of equal and larger than 5, thus it provided a strong support for future studies in the field of passion for studying.

## 5. Conclusions

The present research is one of the first investigations to comprehensively assess the differences in various psychological characteristics between students with a passion for studying and students without one. Our findings indicated that harmonious passion for studying contributed to more favorable psychological characteristics within variables related to studying, mental or somatic health symptoms, and personal resources compared to the lack of passion for studying. Overall, this indicates that harmonious passion for studying has potential health-promoting and health-protecting effects, whereas a lack of passion for studying may lead to adverse effects. Developing a harmonious passion for studying is highly important for students’ functioning.

Also, we demonstrated that using a person-centered approach is highly practical for dividing groups of students based on their passion profiles. Therefore, we believe that assessment of passion for studying within a person-centered approach seems highly valuable in an academic context. We feel that future studies should use longitudinal design to examine the predictive role of passion for studying in anticipating a wide range of psychological states (e.g., anxiety and depression symptoms, student satisfaction) and academic performance (e.g., university dropout and a grade point average).

## Figures and Tables

**Table 1:** Descriptive statistics and internal consistency reliability for the study variables (*n* = 154).

Variables	Cronbach’s Alpha	*M*	*SD*	Min	Max	Skewness	Kurtosis
PS Harmonious passion	0.84	29.53	5.74	10	41	−0.93	1.42
PS Obsessive passion	0.72	15.79	5.37	6	33	0.66	0.27
PS Passion criteria	0.74	4.62	0.97	2.2	6.8	−0.38	−0.39
OLBI Disengagement	0.75	20.63	3.45	11	32	0.31	0.31
OLBI Exhaustion	0.84	22.5	3.94	14	32	0.19	−0.6
GASE General academic self-efficacy	0.82	14.29	3.32	4	20	−1.05	1.36
PHQ-4 Anxiety symptoms	0.82	3.4	1.83	0	6	0.08	−1.23
PHQ-4 Depression symptoms	0.82	2.68	1.93	0	6	0.33	−0.9
PHQ-4 Total score	0.88	6.08	3.51	0	12	0.23	−1.04
GBB-8 Exhaustion	0.71	4.77	1.81	1	8	0.07	−0.69
GBB-8 Gastrointestinal complaints	0.69	3.05	2.21	0	8	0.4	−0.72
GBB-8 Musculoskeletal complaints	0.73	4.03	2.33	0	8	−0.05	−1.01
GBB-8 Cardiovascular complaints	0.58	2.69	2.17	0	8	0.67	−0.09
GBB-8 Total score	0.82	14.53	6.55	1	32	0.35	−0.44
SVS Vitality	0.81	19.1	5.45	5	31	−0.4	−0.24
BRS Resilience	0.85	17.38	4.86	6	28	−0.24	−0.24
SES Self-esteem	0.89	28.42	5.66	11	40	−0.14	0.02
SOP2 Optimism	0.85	4.21	1.36	1	7	−0.3	−0.67
PSS-4 Perceived stress	0.71	11.64	2.85	5	20	0.12	0.16

**Table 2:** Analysis of differences between the psychological characteristics of students without a passion for studying and the psychological characteristics of students with harmonious passion for studying.

Variables	Students without Passion for Studying (*n* = 89)		Students with Harmonious Passion for Studying (*n* = 62)		*t*(149)	*p*	Cohen’s *d*
	*M*	*SD*	*M*	*SD*			
OLBI Disengagement	21.85	3.02	18.66	2.79	6.593	<0.001	1.091
OLBI Exhaustion	23.47	3.6	20.85	3.82	4.286	<0.001	0.709
GASE General academic self-efficacy	13.74	2.97	14.89	3.65	−2.119	0.036	−0.351
PHQ-4 Anxiety symptoms	3.46	1.8	3.29	1.89	0.562	0.575	0.093
PHQ-4 Depression symptoms	2.99	1.87	2.21	1.93	2.485	0.014	0.411
PHQ-4 Total score	6.45	3.46	5.5	3.54	1.644	0.102	0.272
GBB-8 Exhaustion	4.87	1.72	4.5	1.87	1.238	0.218	0.205
GBB-8 Gastrointestinal complaints	3.11	2.18	2.84	2.15	0.763	0.447	0.126
GBB-8 Musculoskeletal complaints	4.1	2.14	3.77	2.54	0.856	0.394	0.142
GBB-8 Cardiovascular complaints	2.79	2.28	2.52	2.05	0.746	0.457	0.123
GBB-8 Total score	14.87	6.23	13.63	6.78	1.156	0.249	0.191
SVS Vitality	17.81	5.54	21.29	4.51	−4.092	<0.001	−0.677
BRS Resilience	16.71	4.49	18.58	5.02	−2.401	0.018	−0.397
SES Self-esteem	27.7	5.45	29.56	5.5	−2.064	0.041	−0.341
SOP2 Optimism	4.06	1.25	4.44	1.44	−1.735	0.085	−0.287
PSS-4 Perceived stress	11.9	2.71	11.18	2.97	1.548	0.124	0.256

**Table 3:** Pearson correlations between the study variables (*n* = 154).

Variables	1	2	3	4	5	6	7	8	9	10	11	12	13	14	15	16	17	18	19
1. PS Harmonious passion	—																		
2. PS Obsessive passion	0.31 ***	—																	
3. PS Passion criteria	0.63 ***	0.44 ***	—																
4. OLBI Disengagement	−0.71 ***	−0.29 ***	−0.56 ***	—															
5. OLBI Exhaustion	−0.55 ***	−0.1	−0.34 ***	0.64 ***	—														
6. GASE General academic self-efficacy	0.23 **	0.09	0.27 ***	−0.18 *	−0.20 *	—													
7. PHQ-4 Anxiety symptoms	−0.22 **	0.06	−0.05	0.26 **	0.44 ***	−0.06	—												
8. PHQ-4 Depression symptoms	−0.24 **	0.01	−0.17 *	0.34 ***	0.48 ***	−0.04	0.75 ***	—											
9. PHQ-4 Total score	−0.25 **	0.04	−0.12	0.32 ***	0.49 ***	−0.05	0.93 ***	0.94 ***	—										
10. GBB-8 Exhaustion	−0.28 ***	0.1	−0.08	0.36 ***	0.55 ***	−0.15	0.54 ***	0.60 ***	0.61 ***	—									
11. GBB-8 Gastrointestinal complaints	−0.12	0.11	−0.04	0.21 *	0.26 ***	−0.13	0.39 ***	0.37 ***	0.40 ***	0.46 ***	—								
12. GBB-8 Musculoskeletal complaints	−0.22 **	0	−0.04	0.23 **	0.35 ***	−0.20 *	0.24 **	0.28 ***	0.28 ***	0.47 ***	0.42 ***	—							
13. GBB-8 Cardiovascular complaints	−0.08	0.13	−0.04	0.18 *	0.27 ***	−0.07	0.37 ***	0.39 ***	0.41 ***	0.49 ***	0.48 ***	0.42 ***	—						
14. GBB-8 Total score	−0.22 **	0.11	−0.07	0.31 ***	0.45 ***	−0.18 *	0.49 ***	0.52 ***	0.54 ***	0.76 ***	0.77 ***	0.77 ***	0.78 ***	—					
15. SVS Vitality	0.45 ***	0.08	0.30 ***	−0.41 ***	−0.47 ***	0.14	−0.35 ***	−0.46 ***	−0.44 ***	−0.37 ***	−0.19 *	−0.21 **	−0.17 *	−0.30 ***	—				
16. BRS Resilience	0.29 ***	−0.09	0.14	−0.24 **	−0.47 ***	0.06	−0.45 ***	−0.40 ***	−0.45 ***	−0.41 ***	−0.27 ***	−0.26 **	−0.37 ***	−0.42 ***	0.38 ***	—			
17. SES Self-esteem	0.06	−0.23 **	0.07	−0.12	−0.27 ***	0.19 *	−0.39 ***	−0.48 ***	−0.46 ***	−0.35 ***	−0.23 **	−0.17 *	−0.21 **	−0.31 ***	0.31 ***	0.37 ***	—		
18. SOP2 Optimism	0.22 **	−0.09	0.14	−0.25 **	−0.39 ***	0.13	−0.43 ***	−0.39 ***	−0.44 ***	−0.35 ***	−0.20 *	−0.13	−0.23 **	−0.28 ***	0.45 ***	0.49 ***	0.63 ***	—	
19. PSS-4 Perceived stress	−0.31 ***	0.08	−0.12	0.31 ***	0.44 ***	−0.08	0.62 ***	0.65 ***	0.68 ***	0.52 ***	0.35 ***	0.35 ***	0.35 ***	0.51 ***	−0.42 ***	−0.47 ***	−0.48 ***	−0.40 ***	—

*Note*. * *p* < 0.05, ** *p* < 0.01, *** *p* < 0.001.

## Data Availability

The raw data supporting the conclusions of this article will be made available by the authors on request.
